# Supplementary material for: The Rac1 homolog CED-10 is a component of the MES-1/SRC-1 pathway for asymmetric division of the C. elegans EMS blastomere
Source: bioRxiv. 2024 May 2:2024.04.04.588162. Originally published 2024 Apr 8. Preprint. [Version 2] doi: 10.1101/2024.04.04.588162 (PMC11030239; doi:10.1101/2024.04.04.588162)
Supplement: 1 [file NIHPP2024.04.04.588162V2-supplement-1.pdf]

bioRxiv preprint doi: https://doi.org/10.1101/2024.04.04.588162; this version posted May 2, 2024. The copyright holder for this preprint (which was not certified by peer review) is the author/funder, who has granted bioRxiv a license to display the preprint in perpetuity. It is made available under aCC-BY 4.0 International license.

Supplemental Fig. 1

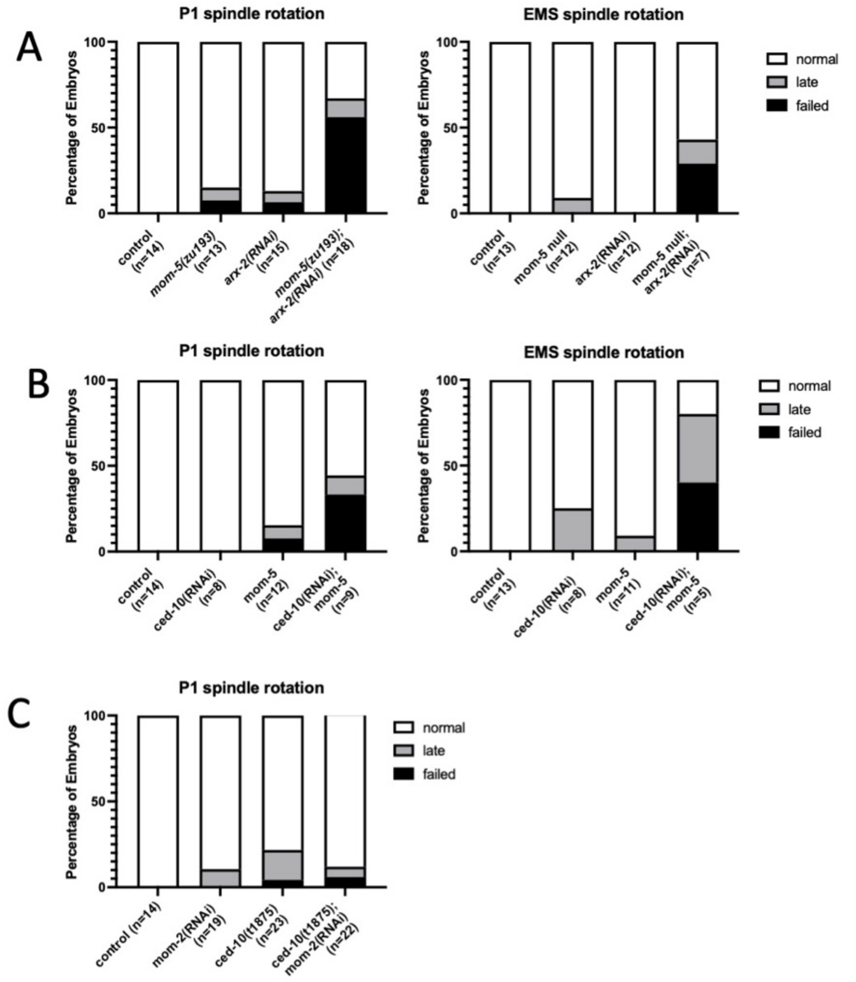

Figure S1. ARX-2 and CED-10 genetically interact with MOM-5(Frizzled) during P1 nuclear rotation. Percentage of scored embryos with normal, late, and failed EMS spindle rotations for the indicated genotypes. (A) Examination of P1 and EMS nuclear rotation in *arx-2; mom-5* double mutants. (B) P1 and EMS nuclear rotation in *ced-10; mom-5* double mutants. (C) P1 rotation in *ced-10; mom-2* double mutants.
